# Advantages of using 3D virtual reality based training in persons with Parkinson’s disease: a parallel study

**DOI:** 10.1186/s12984-019-0601-1

**Published:** 2019-10-17

**Authors:** Imre Cikajlo, Karmen Peterlin Potisk

**Affiliations:** 1 University Rehabilitation Institute, Republic of Slovenia, Linhartova 51, SI-1000 Ljubljana, Slovenia; 2 School of Engineering and Management, University of Nova Gorica, Vipavska 13, SI-5000 Nova Gorica, Slovenia

**Keywords:** Rehabilitation, Virtual reality, Exergaming, Upper extremities, Telerehabilitation, Intrinsic motivation inventory

## Abstract

**Background:**

Parkinson’s disease (PD) is a slowly progressive neurodegenerative disease. There are mixed reports on success of physiotherapy in patients with PD. Our objective was to investigate the functional improvements, motivation aspects and clinical effectiveness when using immersive 3D virtual reality versus non-immersive 2D exergaming.

**Methods:**

We designed a randomized parallel study with 97 patients, but only 20 eligible participants were randomized in 2 groups; the one using 3D Oculus Rift CV1 and the other using a laptop. Both groups participated in the 10-session 3 weeks training with a pick and place task in the virtual world requiring precise hand movement to manipulate the virtual cubes. The kinematics of the hand was traced with Leap motion controller, motivation effect was assessed with modified Intrinsic Motivation Inventory and clinical effectiveness was evaluated with Box & Blocks Test (BBT) and shortened Unified Parkinson’s disease rating scale (UPDRS) before and after the training. Mack-Skilling non-parametrical statistical test was used to identify statistically significant differences (*p* < 0.05) and Cohen’s U3 test to find the effect sizes.

**Results:**

Participants in the 3D group demonstrated statistically significant and substantially better performance in average time of manipulation (group x time, *p* = 0.009), number of successfully placed cubes (group x time, *p* = 0.028), average tremor (group x time, *p* = 0.002) and UPDRS for upper limb (U3 = 0.35). The LCD and 3D groups substantially improved their BBT score with training (U3 = 0.7, U3 = 0.6, respectively). However, there were no statistically significant differences in clinical tests between the groups (group x time, *p* = 0.2189, *p* = 0.2850, respectively). In addition the LCD group significantly decreased the pressure/tension (U3 = 0.3), the 3D did not show changes (U3 = 0.5) and the differences between the groups were statistically different (*p* = 0.037). The 3D group demonstrated important increase in effort (U3 = 0.75) and perceived competences (U3 = 0.9).

**Conclusions:**

The outcomes of the study demonstrated that the immersive 3D technology may bring increased interests/enjoyment score resulting in faster and more efficient functional performance. But the 2D technology demonstrated lower pressure/tension score providing similar clinical progress. A study with much larger sample size may also confirm the clinical effectiveness of the approaches.

**Trial registration:**

The small scale randomized pilot study has been registered at ClinicalTrials.gov Identifier: NCT03515746, 4 May 2018

## Background

### Motor function rehabilitation in persons with Parkinson’s disease

Parkinson’s disease (PD) is a slowly progressive neurodegenerative disease with unknown cause. It usually begins in adulthood at the age between 35 and 60 years. The clinical features of the PD are rigidity (muscle stiffness), bradykinesia (slowness of movement), tremor and postural disorders. PD typically affects the patient’s daily activities, quality of life in all stages of the disease. Patients with PD suffer from significantly decreased coordination and have difficulties with precise movements [1]. Currently degeneration of dopaminergic neurons that triggers changes in the basal ganglia network is mainly treated with levodopa and/or dopamine agonist. With progression of disease patients become less responsive to the medication over time. Finger dexterity is insensitive to dopaminergic treatment because the deficit is related to an intrinsic dysfunction of primary somatosensory cortex (S1), which is not reversible by dopaminergic medication [2]. Furthermore the object manipulation depends on fine coordination of the hand and fingers and cannot be restored by repletion of brain dopamine levels. Dopaminergic therapy can improve intensive movements, but not coordinated movements of the arm, hand, and fingertip forces during reaching and grasping [3].

The outcomes of the 12-week therapy for patients with PD demonstrated improvements of gait, strength, tremor and even motor coordination of upper body. The program consisted of exercises determined by the United Parkinson Foundation and some part of karate training and suggested a pharmacologic therapy in addition to the exercise [4]. The findings related to physical activity are also supported by more extensive study in young healthy adults reporting on importance of aerobic exercise that can temporary reduce inhibition in the motor cortex even in low level physical activity exercises [5]. Furthermore the physiotherapy has become an important part of rehabilitation programs of individuals with PD as patients with PD retain more than ¾ of all activities [6]. The authors report on improving mobility activities that are more related to participation. Consequently, a larger number of patients with PD will require physical therapy in the near future. Balance training has become the most frequently applied physical activity in physiotherapy of patients with PD and is also supported by high-quality evidences for specific therapeutic strategies [7].

However, rehabilitation programs for PD population should not be generalized, but critical and meaningful with targeted tasks, possibly at the patient’s home [8]. Patients with PD can successfully improve their motor capabilities with physical therapy, but the amount of such practice and clinical effectiveness are still unclear. The studies suggest that implicit learning in PD is relatively preserved and motor learning seems to be independent from dopamine-replacement therapy [9]. Therefore the evidences of motor learning are weak and the design of effective rehabilitation protocols is crucial. A good example of a designed treatment can be the constrained movement therapy that effectively improved fine and gross performances of upper extremity in people with PD [10].

### The role of virtual reality and exergaming

Designing target based tasks in a real world is often time consuming and functional changes of such tasks could present an expensive and a spatial problem. Exergames provide several options of changing the virtual environment, task difficulty level, object shapes, colors and consequently adapt the specific task to the user requirements. Immersive virtual reality (VR) can exclude the external disturbing factors and has proven effective in cognitive therapies [11], pain management [12] and motivation of elderly. In persons with basal ganglia-related problem the immersive VR may play an important role in learning of visuomotor coordination [13].

Most of the exergaming and VR active and passive interventions in people with PD target gait and balance, the rest cover also global motor functions, cognitive functions, adherence and activities of daily living [14]. The studies reported on improved motor function used commercially available systems and compared the approach with conventional physiotherapy lasting between 4 and 12 weeks. Kim et al. [15] reported that PD patients were able to use immersive VR during walking without adverse effect. VR may have advantages or similar effect as physiotherapy on stride length, gait and balance; however the evidences were weak due to the small sample sizes. VR passive interventions were even more limited [14]. Another option besides the VR was also a motor imagery therapeutic approach using cognitive functions to improve balance, gait and mobility in people with PD. The study [16] reported on benefits of using both techniques, motor imagery and VR, demonstrated mechanism, provided recommendations for therapy and designated the VR as a promising rehabilitation tool for people with PD. In any case it is necessary to emphasize that safety, feasibility and effectiveness are the most important issues. Feasibility is considered the ability and motivation to play the VR games and effectiveness is referred to the output of clinical tests. The review [17] confirms that most of the studies provided sufficient evidences on feasibility of exergaming and VR for people with PD, but clinical effectiveness required large scale randomized control trials. However the application of commercial games may not be a good option, they often appear too difficult for people with PD to play. Instead the exergames and VR tasks should be tailored towards specific clinical populations and adaptable to the user’s capabilities [17]. An example of such VR approach for balance training was a 3D VR ball catching that required full attention for coordination of eye, hand and foot movement [18]. The authors also reported on improved postural control when using virtual motor rehabilitation system [19] and emphasized that the results could be affected by participants ability of cognitive processing as the immersive VR have impact on visual de-sensation [20].

Precise movements such as writing are often impeded by PD and patients usually complain about upper extremity tremor. Consequently the writing size becomes smaller and smaller, clinically named micrographia [21]. Nonmedical intervention like home upper limb strengthening did not demonstrate significant improvement in the size of handwriting despite of the gain in strength. However, more clinical information could have been provided, if Jebsen’s hand test [22] or Unified Parkinson’s Disease Rating Scale (UPDRS) were carried out. On the other hand activities requiring target based eye and hand movements are significantly affected by changes in timing and kinematics [23] in the early stage of PD. Besides tracking of hand movements and its control in PD population a head mounted 3D VR can provide an alternative to the real world application, particularly in neuroimaging environments [24]. The recent 3D technology can provide more realistic games in tailored approaches to motivate patients [25].

### Objectives of the paper

The majority of the previous studies in PD using immersive or non-immersive virtual environment were focused on gross physical movements supported by commercially available or custom designed exergames and compared with the real world or conventional clinical approaches. To the best of our knowledge, none of the existing approaches compared or put in favor immersive or non-immersive virtual reality in precise small scale movements therapy of patients with PD. Therefore we have developed an application for small scale movements with virtual cubes manipulation conceivably appropriate for the PD population to gain precise movements’ timing and kinematics [26]. The hand movement tracking was based on the infrared cameras (IR) making the assessment more convenient for the participants with PD than using inertial measurement system or opto-electronic 3D assessment system [27]. We carried out a parallel randomized study with 2 groups of participants with PD; one group using immersive VR (3D) and the other non-immersive environment (2D), both receiving 10 additional trainings in 3 weeks. We hypothesized that participants will gain fine motor skills, improvement of functionalities, intrinsic motivation and clinical impact in favor of the immersive 3D technology at unchanged medication plan.

## Methods

### 10Cubes exergaming system

**Fig. 1:**
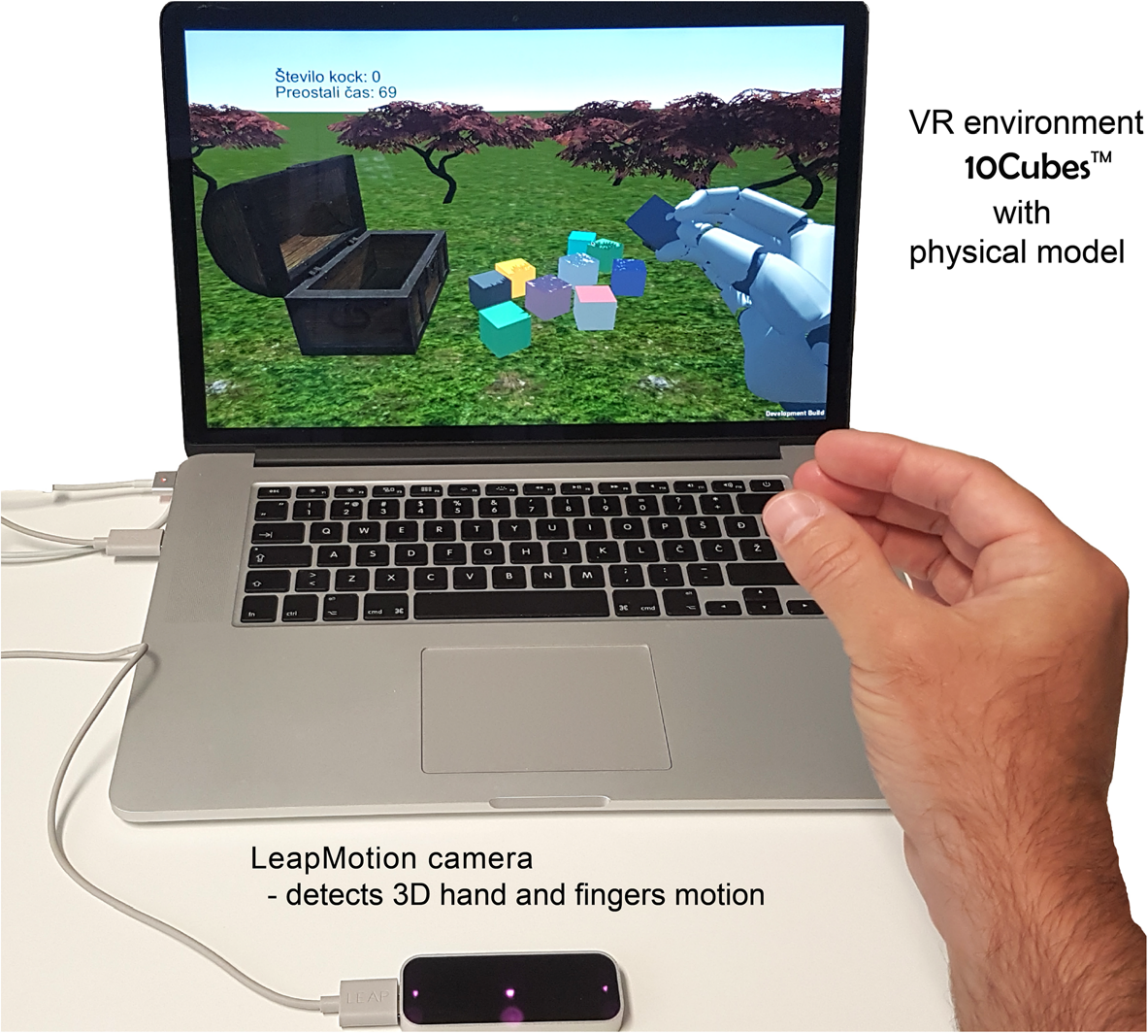
10Cubes system with an infrared camera (Leap Motion Controller) for tracking of hand and finger motion was installed on the laptop computer. The LCD group used such system

The experimental system for the study was based on our developed “10Cubes” exergaming system (Fig. 1). The goal was to have a controllable, tunable and changeable simplified environment, but still similar enough to the Box & Blocks [28]. Therefore we designed a virtual environment with artificial grass and hidden walls and a task with colored cubes that should be picked and placed in the virtual treasure chest by virtual hand with fingers, enabling also a pinch grip. Unity3D (Unity Technologies, CA, USA) software was found suitable for easy and quick modifications of the task (single/double handed, changing objects, color, etc). Particularly for the study we designed a targeted task that required picking 10 multicolored virtual cubes with the same physical model (sizes, weight and material, bounce stiffness) and placing them with a hand one by one in the box, but leaving a room for maneuver. The virtual hand with fingers in the size of the real hand was the user’s avatar and an interface to the task. The user’s real hand and fingers movements were tracked with a small, mouse size 4D camera (2 CCD stereo cameras and 3 infrared LED, Leap Motion Controller, Leap Motion Inc., CA, USA) on the table in front of the sitting person. The Leap Motion Controller (LMC) was connected to the high speed USB 3.0 port of the computer with the suitable graphic adapter (Nvidia GeForce series). The LMC required light calibration or constant light conditions for the pre-calibrated settings. Window blinds were used to assure appropriate unchangeable lighting conditions. The outputs of the LMC were measured coordinates of the hand and each finger segment with an accuracy < 0.5 mm at height between 100 mm and 250 mm [29]. In general the frame rate of the LMC is variable (around 115 Hz) and non-uniform. Resampling with 40 Hz covered the requirements for human motion (Parkinson’s disease resting tremor 3–7 Hz). We used C# and LMC libraries within the MonoDevelop open-source environment to develop on-line data streaming to the Unity3D environment. Initially the game performance was measured by the recorded time and number of successfully placed cubes into the virtual chest. These parameters and the entire kinematics of the hand were recorded on the local computer in ASCII format and sent to the remote server.

**Fig. 2:**
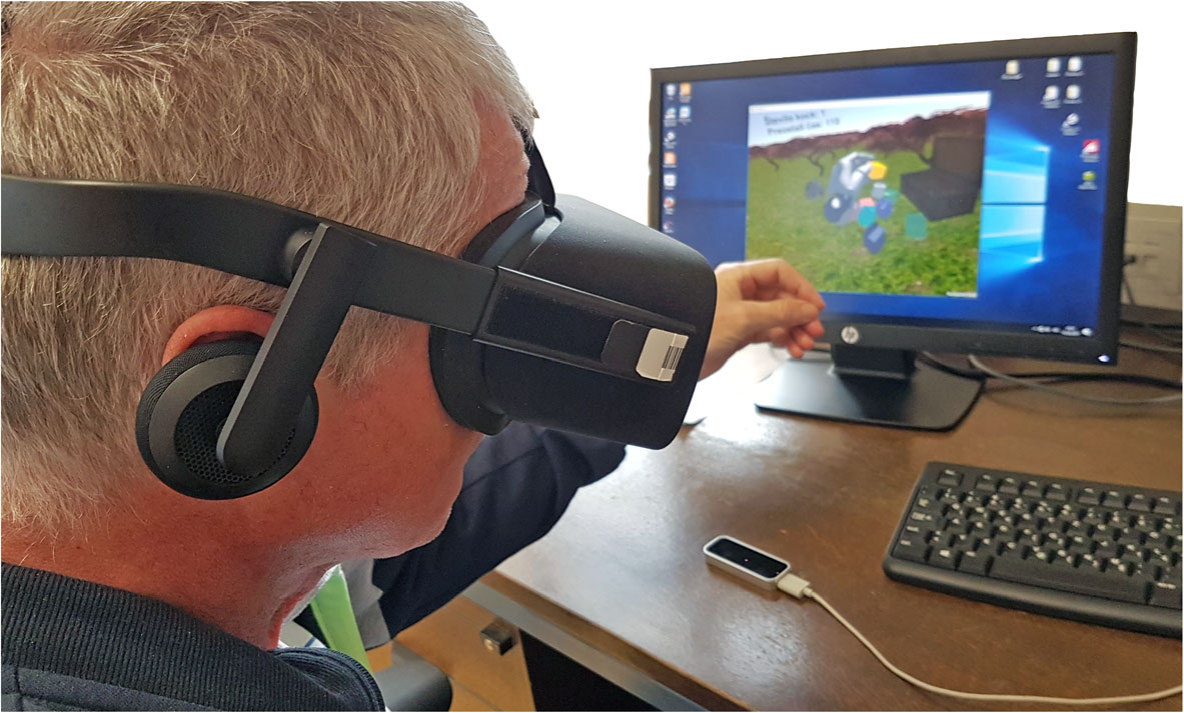
The participants in the 3D group used the 10Cubes settings with the 3D VR Oculus Rift CV1 head mounted device

Two different visualizations were designed; a 2D visualization (Fig. 1) on LCD screen and a 3D visualization (Fig. 2) with the head mounted device (Oculus Rift CV1, Oculus VR, LCC, USA).

### Participants

**Fig. 3:**
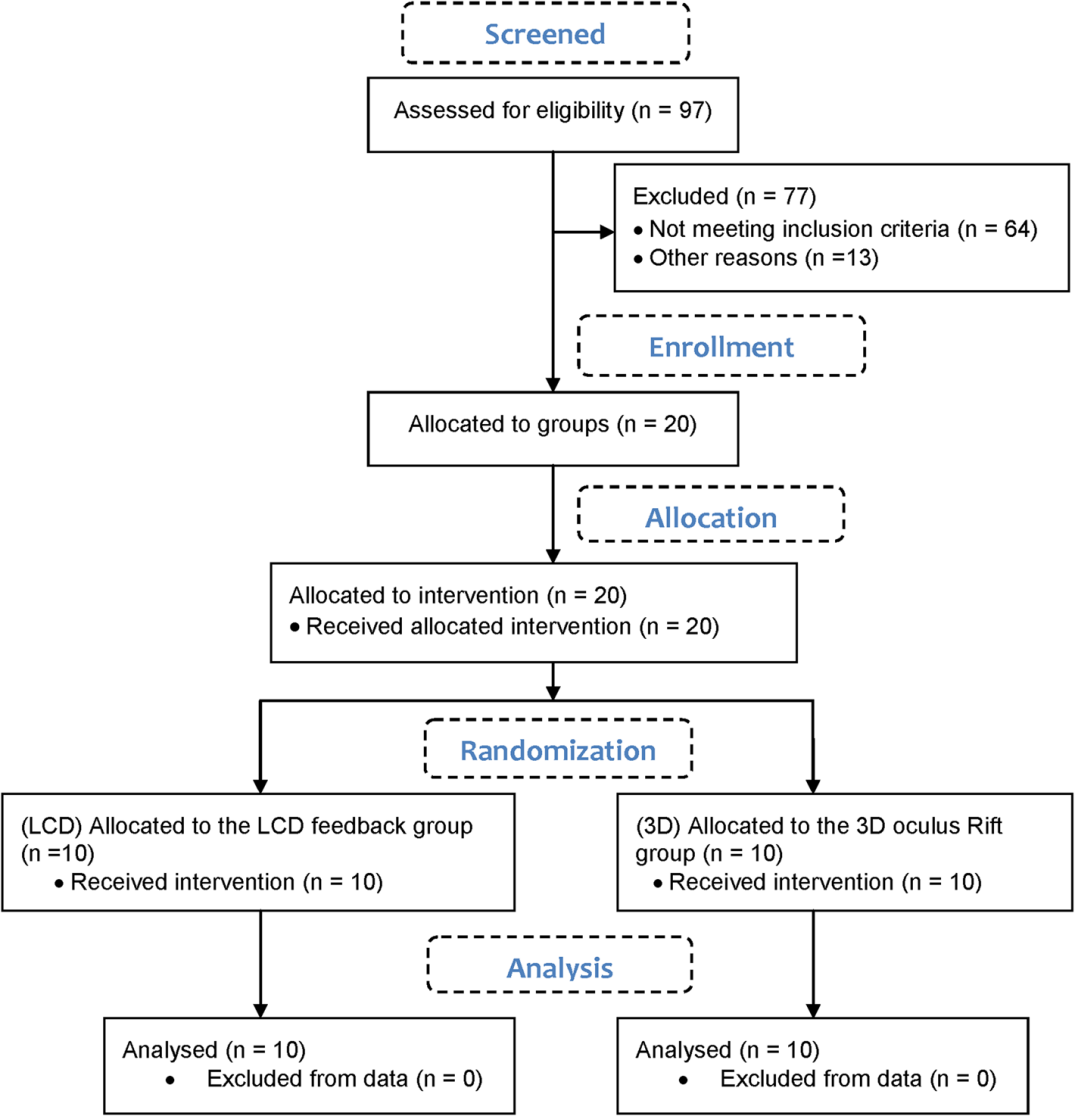
CONSORT Flow Diagram for the parallel randomized study

For the purpose of the study 97 patients with PD were assessed in University rehabilitation hospital, but only 20 were eligible (Fig. 3). According to the responsible physician they met the inclusion criteria: a). PD with functional disorders in upper extremities and minor problems at daily life activities. b). the participants should achieve the level 2–3 in the Hoehn and Yahr Scale [30]. c). ability to follow instructions – Mini Mental State Examination (MMSE) > = 25 [31]. d). no clinically identified stereopsis or history of motion sickness. The immersive equipment was limited to the use of small size glasses (< 14.2 cm), but also allowed focus adjustment for glasses power of less than 4.00 diopters. Participants with more powerful glasses were excluded from the 3D group. All tests were performed by the occupational therapist in the morning when the participants were calm and relaxed (1–2 h after taking medications). The recruited participants (see Additional file 1) were randomized into two groups by drawing lots and allocated to the intervention:

- 10 participants using the 2D equipment (LCD group); 4 males and 6 females, 71.3 ± 8.4 years old, 3 had left side and 7 had right side more affected.
- 10 participants using the 3D equipment (3D group); 5 males and 5 females, 67.6 ± 7.6 years old, 1 had left side and 9 had right side more affected.

The participants in both groups had the PD diagnosed in average 7.1 years ago and had no cognitive impairments that would prevent understanding of the study. Successful randomization had no baseline differences between the groups; gender t(17.99) = 0.43, *p* = 0.67, age t(17.81) = 1.03, *p* = 0.31, affected side t(15.52) = 1.09, *p* = 0.29 and date of diagnosis t(17.62) = 0.07, *p* = 0.94. All sessions were conducted in late 2017 and early 2018 and all the participants allocated to the intervention actually finished the study. The study was approved by local ethics committee and all participants provided a written consent. Participants were free to withdraw at any stage of the protocol without providing a reason.

### Research protocol

All participants in the study had the same neurotherapeutic treatment at the rehabilitation center. The study protocol was the same for both groups despite using different feedback displays for each group. A skilled occupational therapist explained the objectives and the procedure of the study to each of the participants prior to the first session. The participant’s task was to pick and place the 10 virtual cubes in the virtual environment into the open treasure chest using the more affected hand. If both or none of the hands were affected, then the participant would have used his/her dominant hand. When the cube ended up in the open chest, the cube disappeared and the counter increased. However, the cube might have collided with the walls and bounced back to the virtual lawn or dropped out of the hand. Such event did not change the visible counter, but was still recorded. Each participant had 120 s of time available, but could finish the task earlier by putting all the 10 cubes in the chest correctly. The participants were seated on the wooden, safe, but comfortable chair, having a computer/laptop screen in front of them or wearing head mounted display. The LMC was placed on the table in front of the participant in both cases. Usually the occupational therapist started the application, rarely the participant, even if proficient with computers.

The study comprised of 10 sessions within the 3 weeks in two groups. Each session lasted up to 30 min, enough time to finish the VR task 5 times. Short breaks of 1–2 min between the trials were compulsory. The occupational therapist was present all the time and would have offered physical (safety, sitting posture correction and set-up of the immersive equipment) or cognitive assistance (encouraging the patient, reading and providing instructions and communication with the operator or technical support), if assistance was needed.

### Data assessment

#### Hand temporal and spatial parameters

##### Manipulation time of virtual objects

The total time from the first touch with the virtual cube to the last cube inserted in the virtual chest was calculated from stored data by the C# function – *time from first touch to end* (TfFTtE). Each event in the virtual environment (touch with the cube, releasing the cube, un/successfully inserted cube in the chest, identity number of the cube) was recorded with a timestamp additionally to the kinematics of the hand and the fingers (3D position of the center of the segment). The function merged the data of cube ID and timestamps at touch with the cube and calculated the manipulation time of each cube. The *average time of manipulation* (AToM) was determined by the sum of the partial manipulation times divided by the number of trials. The *total number of trials* (TNoT) was given by the sum of all, even partial movements of the virtual cubes. However, the *number of inserted cubes* (IB) was the sum of all successfully placed cubes in the virtual chest.

##### Average tremor indicator

The estimation of a tremor or irregular shaking of the hand required a motion trajectory analysis during the manipulation of the cubes in the virtual environment. We defined the tremor as an irregular fluctuation of the hand position in x, y and z directions and we were looking for the sudden (high frequency < 0.5 s) changes of the direction of movement. Each sudden change of direction of movement (for > 0.5 cm and < 1 cm) increased the tremor indicator parameter; + 1 for a single direction and + 3 for all three axis directions. The obtained value was divided by the number of axes (3) to determine the *average tremor indicator* (ATI), which would be in ideal conditions equal to 1:

$$ {ATI}_{x,y,z}=\frac{1}{N}\sum \limits_{t_{grab}}^{t_{drop}}\left( tremor\ {indicator\ parameter}_{x,y,z}\right) $$

where the sum of all cubes manipulation tremor indicator was divided by the number of trials (N). The ATI was expected between 10 and 20 for the person with PD. Values < 10 were considered low and < 5 extra low and > 20 a high average tremor index, corresponding to the patients’ data of the UPDRS III for upper limb.

#### Assessment of motivation

The participant’s experiences with the 10Cubes were evaluated with the Intrinsic Motivation Inventory (IMI) [32] immediately after the daily session in both groups. The IMI has also been used in previous studies [33] with virtual environment for motor rehabilitation and our modification is given by 8 statements:

- Q1 I have made a lot of efforts to play the game
- Q2 I think it’s a good game for me.
- Q3 The game seemed very interesting to me.
- Q4 I did my best.
- Q5 During the playing I was very tense.
- Q6 I am satisfied with my result.
- Q7 Playing was fun.
- Q8 During the game I felt under pressure.

and the participants were asked to rate each question/statement from 1 to 7 on the 7-point Likert scale, where 1 indicated the total disagreement and 7 full agreement with the particular statement. The participants rating applied to the current session and they were not presented their previous ratings. The statements were transformed into four measure scales: interest/enjoyment (Q3 + Q7), effort/importance (Q1 + Q4), perceived competence (Q2 + Q6) and pressure/tension (Q5 + Q8). Each of the measure scales has a possible range of points between 2 and 14.

#### Clinical tests

We have additionally checked the effectiveness of the virtual rehabilitation training with the most frequently used clinical tests in PD, the UPDRS [34] and Box & Blocks (BBT). However, only the motor function part of the UPDRS directly related to the upper limb’s function was applied (hand movements L & R, pronation/supination L & R, postural tremor of hands L / R, kinetic tremor of hands L & R). The possible range of points was reduced to an interval between 0 and 24, where 0 means no disability and 24 means total disability. The BBT is the occupational therapist’s tool to evaluate unilateral gross manual dexterity. The goal is to place as many wooden cubes as you can to the other compartment in 60 s.

The clinical tests for both groups of participants were carried out by the skilled occupational therapist 1 day prior and maximum 1 day after the VR task training in the rehabilitation center.

### Data analysis

Matlab (MathWorks, Natick MA, USA) was used as a main tool for raw data extraction, filtering and analysis (see Additional files 2 and 3). All missing data were interpolated and non-numbers were eliminated. Matlab Statistical Toolbox and its Bartlett’s test were used to check the data for normality and equality of variances. If the data fails the homogeneity test, we would apply a non-parametric Friedman’s test for intervention and time effect. However, the data were also unbalanced and we applied the equivalent of the Friedman’s test, the Mac-Skilling non-parametric test [35]. The significance level was set to *p* = 0.05. The effect size was measured by the Cohen’s U3 index [36] identifying the substantial differences between the samples. The U3 index defined the overlap of the two normal distributions. If the U3 is much greater or smaller than 0.5 the effect would considered large (0 < U3 < 0.2 and 0.8 < U3 < 1), but when equal to 0.5 then U3 would show no effect.

Spatio-temporal performance of the hand during the pick and place task was used to examine the differences between the sessions. The mean values and standard deviations (SD) were calculated for TfFTtE, AToM, IB, TNoT, ATI, ATIpS across the participants for daily sessions and presented for each group. Substantial differences between the 1st and the last session were particularly examined using the Cohen’s U3 index. Interaction between choosing different equipment and training time (group-by-time effect) was examined by the Mac-Skilling non-parametric test for unbalanced data (*p* = 0.05).

The differences in motivation prior to and post training in virtual environment for all four IMI’s measure scales were determined by Cohen’s U3 index in both groups. Additionally we checked the statistically significant differences in the above mentioned scales across the groups by Mac-Skilling non-parametric test for unbalanced data (*p* = 0.05).

Clinical tests BBT and the modified UPDRS for upper limb were expected to demonstrate the effectiveness of the intervention. We presented the median values assessed prior to and after the sessions and indicated the 25th and 75th percentiles with whiskers at 1.5 times the interquartile range. The expected substantial differences in mean values for intervention were checked by the Cohen’s U3 index. Additionally, the statistical interaction between the groups and the time factor were examined by the Mac-Skilling test.

## Results

### Movement analysis

**Fig. 4:**
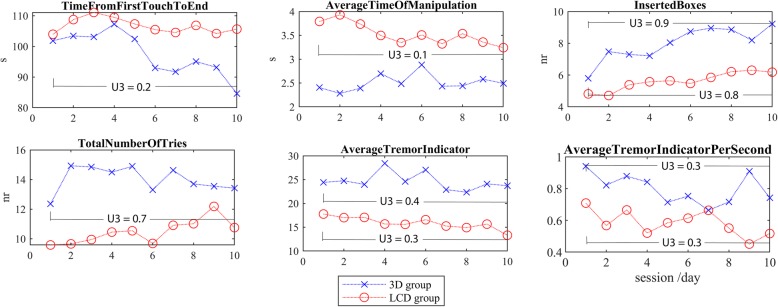
The spatio-temporal parameters of the training performance in the LCD and the 3D group calculated for each of the 10 sessions. The differences between the means of the 1st and last session are presented by the Cohen’s U3 index

The participants from the 3D group managed to put 9.2 cubes on average in the chest before the time elapses (< 120 s) in the last session; on average in 84.6 s (Table 1). This was indeed much faster than in the first session (101.9 s). Namely these participants were also faster than the LCD group (2.40 vs 3.79 s) at manipulation of each cube (Fig. 4). This often resulted in inaccuracy, lost cubes and consequently increased number of attempts (13.4 vs 12.4). We found substantial differences (U3 < 0.2 or U3 > 0.7) between the first and the last session for each group of participants (Table 1). Particularly, the TfFTtE has decreased from 101.9 to 84.6 s (U3 = 0.2) due to successful completion of the task (IB). On the other hand the LCD group systematically reduced the AToM (U3 = 0.1) and increased the number of successfully inserted cubes (IB, U3 = 0.8). The tremor index has marginally decreased (from 17.8 to 15.5), in particular the LCD group demonstrated improvement by the Cohen’s U3 index (Table 1).

**Table 1:** The results of the performance at 1st session (S1) and last session (S10); TfFTtE (Time from first touch to the end), AToM (Average time of manipulation), IB (Inserted boxes), TNoT (Total number of tries), ATI (Average tremor indicator), ATIpS (Average tremor indicator per second) The Mac-Skilling non-parametric test was used to test the statistical differences (group x time, *p* < 0.05). The Cohen’s U3 coefficient demonstrated substantial differences in effect size between S1 and S10

		LCD group			Cohen’s		3D VR group			Cohen’s	Mac-Skilling
	S1		S10		U3	S1		S10		U3	p
	mean	sd	mean	sd	[CI]	mean	sd	mean	sd	[CI]	
TfFTtE	103,98	11,97	104,19	12,54	0,4 [0,1-0,8]	101,87	24,56	84,60	29,54	**0,2** [0,0-0,7]	0,669
AToM	3,79	1,72	3,36	1,36	**0,1** [0,0-0,9]	2,40	0,50	2,49	0,51	0,6 [0,2-0,9]	0,009*
IB	4,81	2,27	6,31	2,87	**0,8** [0,2-1,0]	5,80	2,84	9,22	1,24	**0,9** [0,6-1,0]	0,028*
TNoT	9,58	3,54	12,19	3,44	0,5 [0,2-1,0]	12,36	4,64	13,42	2,91	**0,7** [0,2-1,0]	0,181
ATI	17,77	8,23	15,52	5,45	**0,3** [0,0-0,8]	24,42	8,41	23,72	8,53	0,4 [0,0-0,9]	0,002*
ATIpS	0,71	0,47	0,45	0,25	**0,3** [0,0-0,8]	0,94	0,37	0,74	0,18	**0,3** [0,0-0,8]	0,001*

*statistically significant differences (*p* < 0.05)

substantial differences (bold)

The changes in performances over time were different for the groups – the differences were statistically significant (*p* < 0.05) for AToM (*p* = 0.009), IB (*p* = 0.028) and tremor (*p* = 0.002, *p* = 0.001, respectively, Table 1).

### Intrinsic motivation inventory

**Fig. 5:**
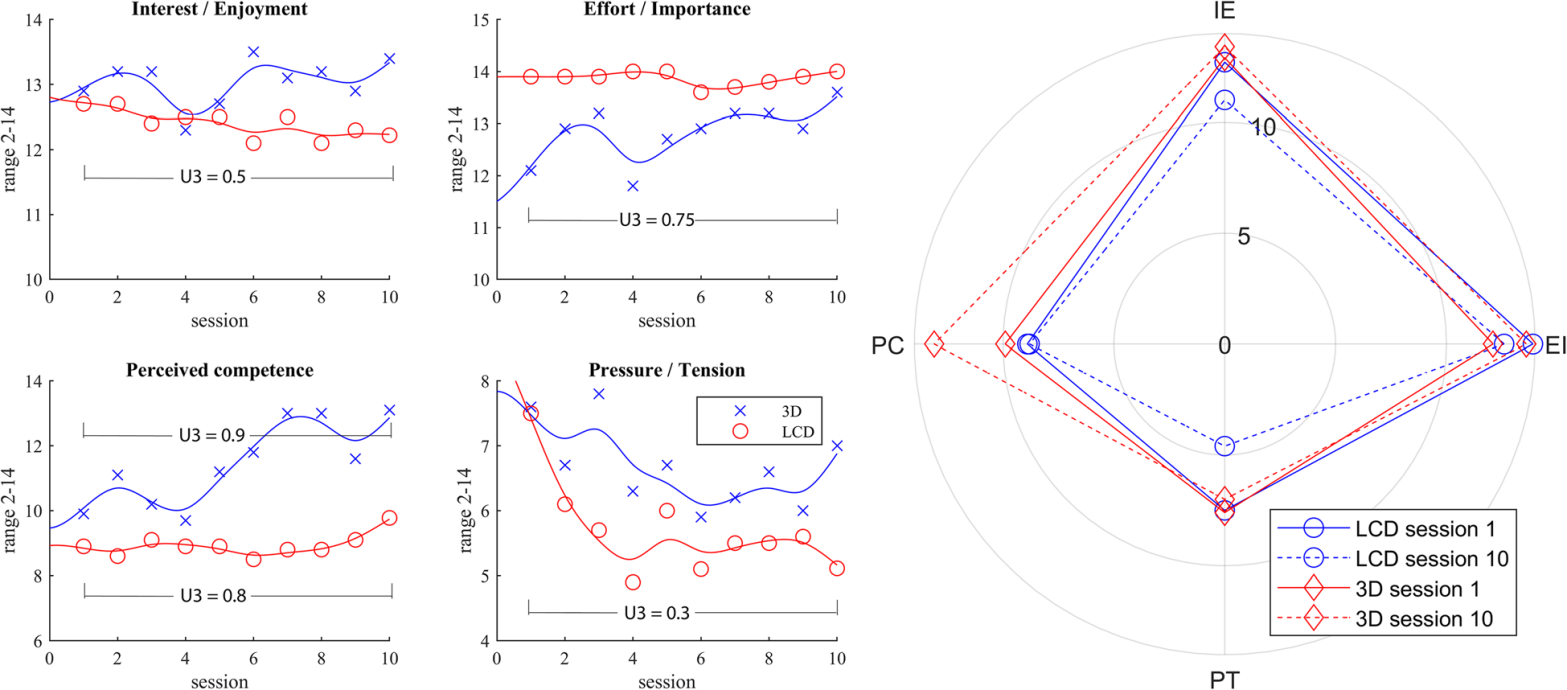
The changes in IMI measure scales in the LCD and the 3D group for each of the 10 sessions. Interpolation with smoothing spline polynomials is presented. The differences between the means of the 1st and 10th session are presented by the Cohen’s U3 index. The polar plot demonstrates that the changes of the IMI measure scales in the LCD group were negative and the in 3D group positive or remain equal

Both participating groups demonstrated substantially significant differences between the 1st and the last session (Table 2) for the “perceived competences” measure scale (LCD group 8.9 to 9.8, U3 = 0.8, 3D group 9.9 to 13.1, U3 = 0.9). Furthermore, the differences between the groups and the two sessions were statistically significant (p = 0.037). However, none of the other measure scales reported on differences between the groups after the session; the 3D VR group demonstrated substantially more in effort/importance measure scale and the LCD group felt substantially less pressure/tension (Fig. 5) in the last session. Despite the measure scale reported on rise of interest/enjoyment in the 3D group and decline in the LCD group (in Fig. 5 upper left) the differences were rather negligible (U3 = 0.5, *p* = 0.995).

**Table 2:** The Cohen’s U3 coefficient demonstrated substantial differences in effect size between 1st and last session for IMI measure scales

	LCD group	3D VR group	
	Cohen’s U3 [CI]	Cohen’s U3 [CI]	Mac-Skilling
	session 1 vs 10	session 1 vs 10	p
Interest / Enjoyment	0,5 [0,1-0,8]	0,5 [0,4-0,9]	0,995
Effort / Importance	0,5 [0,5-0,5]	**0,8** [0,5-0,9]	0,418
Perceived competence	**0,8** [0,2-1,0]	**0,9** [0,5-1,0]	0,037*
Pressure / Tension	**0,3** [0,1-0,7]	0,5 [0,0-1,0]	0,422

*statistically significant differences (*p* < 0.05)

substantial differences (bold)

The polar plot in the Fig. 5 demonstrates that the 3D group in fact increased the perceived competences and interest/enjoyment at less pressure/tension from session 1 to session 10. The group also raised the effort/importance (U3 = 0.8). On contrary the LCD group did not gain but rather lose interest/enjoyment at unchanged effort and still managed to increase their perceived competences at significantly decreased pressure/tension.

**Fig. 6:**
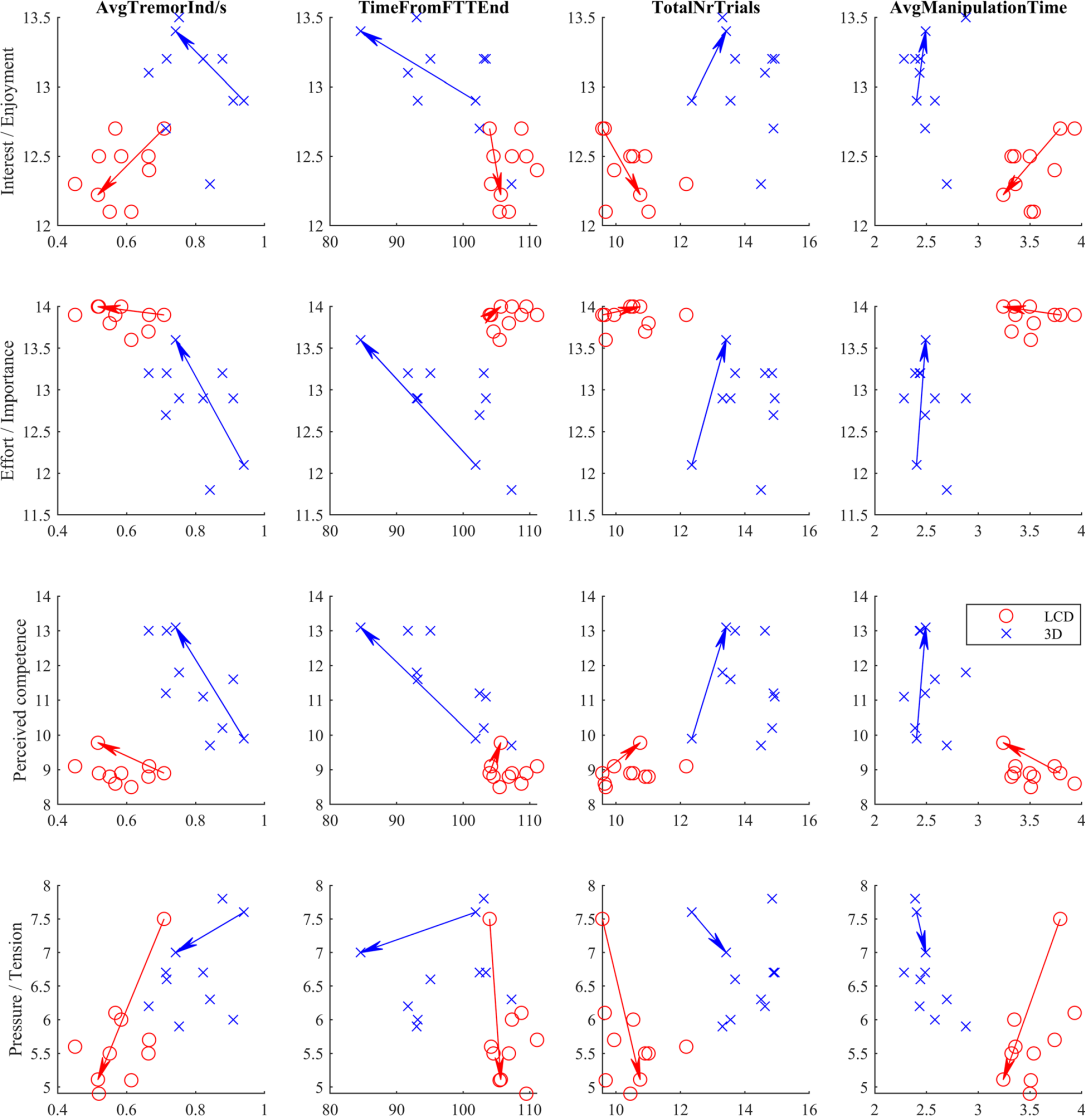
The relationship between the IMI measure scales and the kinematic and game parameters for each of the 10 sessions. The arrows present the change from the 1st to the final session. The lower pressure/tension score in the LCD group resulted in the slower virtual cube manipulations, but fewer attempts resulted in more accurate movements with a lower tremor index

The Fig. 6 shows kinematic performance in relation to the participant’s motivation for each particular session. The participants in the LCD group were putting a lot of effort (based on IMI effort score) into the game, almost without pressure and tension. This corresponds to slower performance, but with fewer attempts. On contrary the 3D group demonstrated higher interest to achieve good results and put much pressure on them. Thus many failed attempts were unavoidable. However, the group was still faster in performance and estimated higher perceived competencies.

### Clinical outcomes

**Fig. 7:**
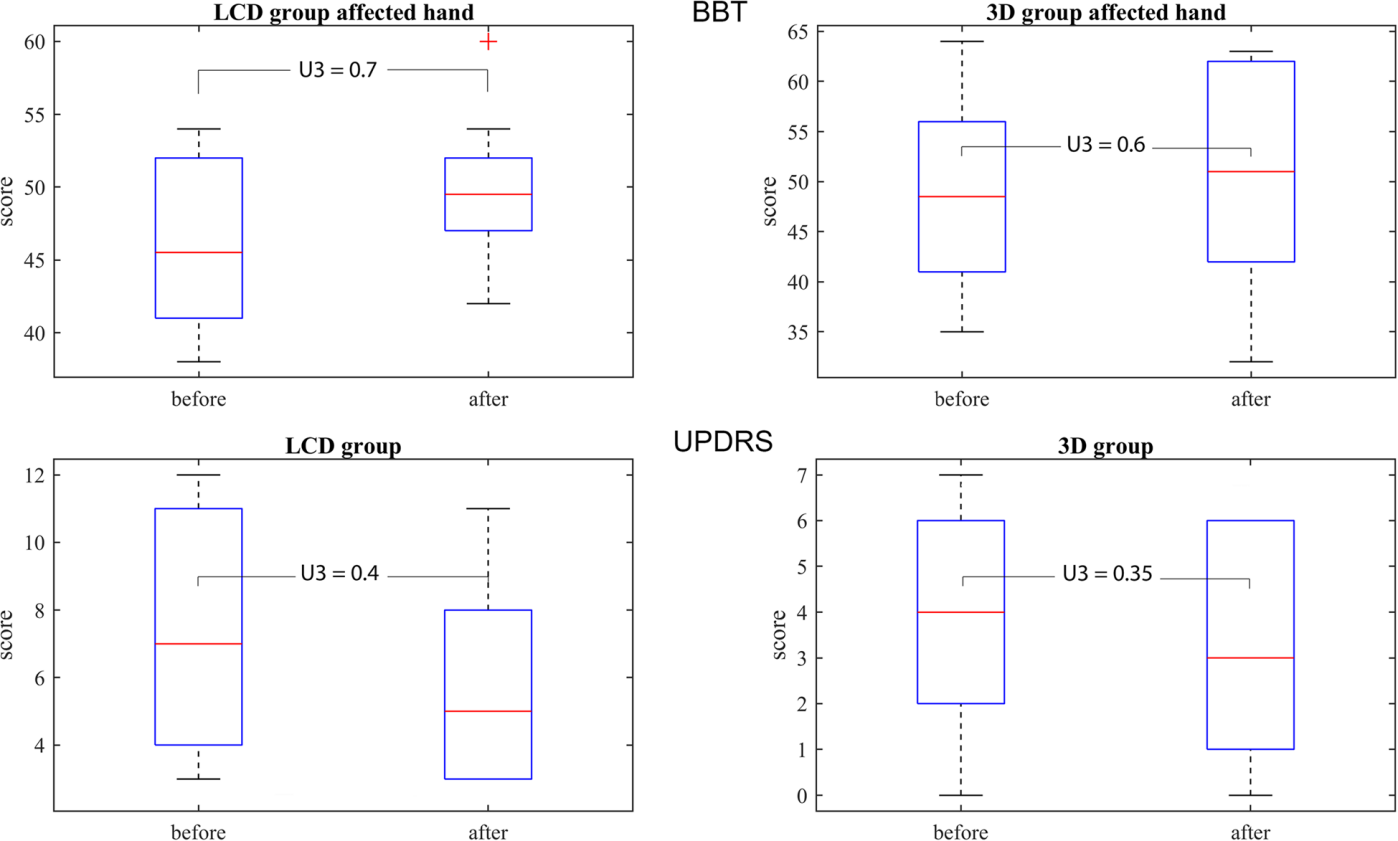
Median values, 25th and 75th percentile and 1.5 times whiskers of the BBT (upper) and UPDRS III for lower limb (lower) data for LCD and 3D groups before and after the virtual environment sessions. Both clinical tests demonstrated improvement in terms of mean values. The effect sizes are presented by the Cohen’s U3 index

The mean value of the BBT increased from 46.30 SD 5.50 to 49.6 SD 5.87 cubes in the LCD group and from 48.50 SD 9.37 to 50.10 SD 9.97 for the affected hand in the 3D group after the sessions. The Fig. 7 shows the median values, the 25th and 75th percentile and the whiskers 1.5 times the interquartile range. The effect sizes are presented with Cohen’s U3 and 95% confidence interval (CI): U3 = 0.7 CI [0.30–0.95] and U3 = 0.6 CI [0.15–1.00] for the LCD and for the 3D group, respectively (in Fig. 7 above). The Mac-Skilling non-parametrical test for unbalanced data cannot confirm statistically significant differences between the groups before and after the sessions (*p* = 0.285).

The UPDRS for upper limb demonstrated in average a lower score after the sessions for the LCD group (from 7.40 SD 3.44 to 5.80 SD 2.82 points) and also for the 3D group (from 3.90 SD 2.26 to 3.30 SD 2.24 points). The Fig. 7 below shows the median values and the Cohen’s U3 = 0.4 CI [0–0.75] and U3 = 0.35 CI [0.05–0.85] for LCD and 3D groups, respectively. However, the differences between the groups before and after the sessions were not statistically significant (*p* = 0.2189).

## Discussion

### Functional and motivational aspect

Participants demonstrated high interest and enjoyment score at the beginning of the sessions in both groups. The 3D group’s interest/enjoyment score rose until the end of the sessions and the members of the 3D group gave the task high priority, managed to keep the average manipulation time of the cube low and thus had enough time to correct mistakes and significantly increase the number of inserted boxes until the end of the sessions. This resulted in much shorter total task time (TfFTtE), but also increased pressure/tension score. Namely the higher pressure/tension in the 3D group affected the preciseness and the total number of attempts was evidently higher. But the group members achieved the goal score and finished the task much faster than at the beginning of the sessions. The level of pressure/tension score corresponded to the higher level of average tremor index. The tremor detection has been a subject of research in Parkinson’s disease [37] for various reasons, but one of them remains objective evaluation at home or the telerehabilitation services where the number of clinical tests shall be reduced. Such indicator may complement an online pressure/tension questionnaire in telerehabilitation service and thus provide a valuable information on cause of the participant’s tremor and its level [24].

The interest/enjoyment score in the LCD group was gradually decreasing at almost constant level of the effort/importance parameter as reported by the IMI. The group patiently indicated precision and reliability at cube manipulation and gradually decreased the average time of the cube manipulation. The total time of the task (TfFTtE) remained in the same range, but the group managed to increase the final score (inserted boxes) with low number of total trials and low pressure/tension score. The members of the LCD group reported on slightly higher perceived competences at the end of the sessions and we objectively estimated lower average tremor index.

Perhaps the LMC may appear a less reliable and cheap equipment, but it can be efficiently used for the kinematic evaluation and the tremor estimation where a required accuracy is rather low [38]. We still need to take in consideration that distinguishing essential tremor from PD is challenging, particularly in the early stages of the disease. Despite the tremor appears in different frequency ranges even the accelerometer and electromyography based quantitative analysis require larger studies to develop a gold standard [39].

### Clinical aspect

The physical therapy in PD is usually evaluated by Berg balance scale, Five Time Sit to Stand (FTSTS), 6 min walking test and Freezing of gait (FOG), most of the tests relevant to the gait and balance only [6, 19]. Only few publications tried to justify the therapy or evaluate the modern technology and/or specific fine motor movements with UPDRS III [18, 37, 38, 40]. The pick and place tasks are often considered essential for the improvement of daily living tasks. Most of the daily living tasks are associated with the ability to dynamically manipulate objects and coordinate the movements of hands, arms, fingertips and control grasping. Dynamical handling of objects is usually affected in persons with PD, in particular the scaling and coordination aspect of motor control [1]. The authors [3] used the Phantom haptic device with specifically designed tasks and demonstrated that dopaminergic therapy significantly improved the intensive movements, but not the coordinative aspects of movements. On the other hand the virtual reality applications require coordination of visual and motor control to perform an accurate movements [23]. Perhaps the best option would be a combination of medication and regular physiotherapy [14], with VR application as an additional tool to enable more targeted, modifiable and controlled physiotherapy without additional physically strenuous activity of the physiotherapists. The motor performance of persons with PD can be improved either with virtual objects or real physical objects moving [41].

We also found positive effects of the virtual rehabilitation in all 20 participants improving or at least keeping the same score at the UPDRS III motor, UPDRS III for upper limb test and BBT clinical tests. The minor but positive changes in the UPDRS III for upper limb were more or less similar for both visual interfaces. However, the BBT test revealed medium effect size for both groups indicating that such therapy improves performance of the upper limb fine movements [10]. Comparable outcomes were identified with the BBT even the training protocol was not longer than 3 weeks and comprised half of the sessions.

### (Dis)advantages of 3D equipment vs 2D exergaming

The participants using the immersive 3D equipment were more motivated (enjoyment/interest) than the members of the LCD group, particularly at the final sessions. The first group’s interest/enjoyment score was 10% higher at the end, but practically the same prior to the sessions. As the effort/importance rose gradually in the 3D group, the group perceived more competences than the LCD group. Most of the participants successfully accomplished the task earlier despite of several trials. However, the strong desire to finish the task on time may have caused the increase of the pressure/tension score in the 3D group in the final sessions. Additionally we have noticed the rise of the average tremor indicator in the penultimate session. We found the 3D group performance more efficient and better in terms of kinematic and game parameters comparing to the LCD group, but at the expense of pressure/tension. However, in terms of clinical tests both protocols provided comparable positive outcomes. There might have been also progress in visual-motor control as persons with PD experience visual de-sensation [20]. However, this was only indirectly measured and reported with kinematics parameters.

IMI may not necessarily be the best instrument for identification of differences between groups performing similar or the same exercises [42]. Some standard questions appeared tricky for some participants, like how they think they performed the game – their answers were “I did my best”, and at the same time “My results were poor”. Occupational therapy usually measures the motivation with questionnaires, but this may not always be the case. Some researches on cognitive rehabilitation in the past eliminated the time constrain to release the stress and measured physiological changes that are indirectly related to the person’s emotional state or reaction [43]. Such measurement might be more objective and useful for adaptive task control, but confronts with the exhausted patients and the measurements may become controversial.

The study was limited to the Oculus Rift CV1 HMD that enabled the participants with eyeglasses to use the HMD with adjustable lens without their spectacles and use stereo headphones. Instead of music we provided singing of birds to avoid the rhythmic movements [44]. Such equipment enabled the participants augmented perception; focus entirely on the task without any external sensory inputs. We primarily focused on adaptive controlled difficulty level according to the participants’ ability to perform the VR task, but in the study we kept the same difficulty level without changing the size, color and bouncing materials of the cubes. The use of commercial games was primarily excluded as most of them are too complex and difficult for the participants [17].

Our findings are based on a reasonably large entry sample size, but still limited number of participants to make clinical conclusions. However, most of the existing studies using virtual rehabilitation are facing the same problem [14].

## Conclusion

Regardless of the visual technology used in the study the participants have improved fine motor skills of the upper limb in terms of clinical tests and kinematic measures. Some of the positive outcomes surely corresponded to the higher motivation of the participants. Those using the 3D visual equipment increased their enjoyment/interest motivation score that resulted in faster and more efficient functional performance, apparently due to the augmented perception. This was not possible with the 2D equipment and presumably the LCD group was gradually losing their interest and consequently putting less effort into task performance. But the LCD group obviously felt more relaxed, making fewer mistakes than the 3D group. For those who found the pick and place the cubes motivating but had problems with the difficulty level, we are planning to use objective motivation measurement and dynamic difficulty adaptation to make the training more challenging and therefore increase the interest/enjoyment level.

The immersive 3D equipment may not be appropriate for every person with PD; in particular users with glasses may experience problems with the correct adjustment of the equipment, visual discomfort, fatigue or even transient myopia.

## Supplementary information

**Additional file 1.** Participants’ data

**Additional file 2.** Data table of 3D group for Matlab

**Additional file 3.** Data table of 2D group for Matlab

## Data Availability

Anonymized data generated or analyzed during this study are included in this published article [and its supplementary information files].
